# Comprehensive Analysis and Verification of the Prognostic Significance of Cuproptosis-Related Genes in Colon Adenocarcinoma

**DOI:** 10.3390/ijms252111830

**Published:** 2024-11-04

**Authors:** Yixiao Gu, Chengze Li, Yinan Yan, Jingmei Ming, Yuanhua Li, Xiang Chao, Tieshan Wang

**Affiliations:** 1 School of Traditional Chinese Medicine, Beijing University of Chinese Medicine, Beijing 100029, China; 20220931101@bucm.edu.cn (Y.G.); chengze_li163@163.com (C.L.); 20230931096@bucm.edu.cn (Y.Y.); 2 School of Chinese Material Medical, Beijing University of Chinese Medicine, Beijing 100029, China; 20220221172@bucm.edu.cn (J.M.); 20220221002@bucm.edu.cn (Y.L.); 3 Beijing Research Institute of Chinese Medicine, Beijing University of Chinese Medicine, Beijing 100029, China

**Keywords:** colon adenocarcinoma, cuproptosis, prognosis, tumor microenvironment, drug sensitivity

## Abstract

Colon adenocarcinoma (COAD) is a frequently occurring and lethal cancer. Cuproptosis is an emerging type of cell death, and the underlying pathways involved in this process in COAD remain poorly understood. Transcriptomic and clinical data for COAD patients were collected from The Cancer Genome Atlas (TCGA) and Gene Expression Omnibus (GEO) databases. We investigated alterations in DNA and chromatin of cuproptosis-related genes (CRGs) in COAD. In order to identify predictive differentially expressed genes (DEGs) and various molecular subtypes, we used consensus cluster analysis. Through univariate, multivariate, and Lasso Cox regression analyses, four CRGs were identified. A risk prognostic model for cuproptosis characteristics was constructed based on four CRGs. This study also examined the association between the risk score and the tumor microenvironment (TME), the immune landscape, and drug sensitivity. We distinguished two unique molecular subtypes using consensus clustering analysis. We discovered that the clinical characteristics, prognosis, and TME cell infiltration characteristics of patients with multilayer CRG subtypes were all connected. The internal and external evaluations of the predicted accuracy of the prognostic model built using data derived from a cuproptosis risk score were completed at the same time. A nomogram and a clinical pathological analysis make it more useful in the field of medicine. A significant rise in immunosuppressive cells was observed in the high cuproptosis risk score group, with a correlation identified between the cuproptosis risk score and immune cell infiltration. Despite generally poor prognoses, the patients with a high cuproptosis risk but low tumor mutation burden (TMB), cancer stem cell (CSC) index, or microsatellite instability (MSI) may still benefit from immunotherapy. Furthermore, the cuproptosis risk score positively correlated with immune checkpoint gene expression. Analyzing the potential sensitivity to medications could aid in the development of clinical chemotherapy regimens and decision-making. CRGs are the subject of our in-depth study, which exposed an array of regulatory mechanisms impacting TME. In addition, we performed additional data mining into clinical features, prognosis effectiveness, and possible treatment medications. COAD’s molecular pathways will be better understood, leading to more precise treatment options.

## 1. Introduction

One of the most prevalent malignancies globally, colorectal cancer (CRC) is steadily on the rise in both incidence and mortality rates, with colorectal adenocarcinoma (COAD) representing the most common subtype [1]. Despite advancements in medical treatment, the quality of life and prognosis for COAD patients remain largely unchanged, with a persistently low 5-year survival rate [2]. Efficacious COAD therapy is challenging because of late detection, and early biomarker screening helps individuals with COAD have improved prognoses [3]. Consequently, it is essential to investigate and build reliable prognostic models. For metalloenzymes/proteins such as superoxide dismutase 1 (SOD1) and lysyl oxidase, copper is an essential cofactor. There are several human disorders that may be aggravated or worsened by an unbalanced copper ion homeostasis, including cancer. Copper levels in cancer patients’ blood and tumor tissue are substantially greater than those in healthy people, according to the literature [4]. Tumor angiogenesis may be promoted by copper, which has been linked to cancer [5]. A novel mechanism for cell death called cuproptosis has just been found [6]. Copper ions interact with the lipoylation components of the tricarboxylic acid cycle during mitochondrial respiration, leading to protein toxic stress and cell death. Twelve genes related to cuproptosis have been identified: seven promoting genes (FDX1, LIAS, LIPT1, DLD, DLAT, PDHA1, PDHB), three inhibiting genes (MTF1, GLS, CDKN2A), and two key copper transporters (ATP7B, SLC31A1). A change in tumor metabolism regulates energy metabolism, including Warburg effects (glycolysis and TCA) and fatty acid and glutamate metabolism, to promote cell growth and proliferation [7,8]. At the same time, the metabolites produced by cancer cells can influence immune cells, changing the TME [9]. As a result, enhancing tumor cell immune escape has the potential to impact therapy outcomes. Understanding the connection between the TME and CRGs could inspire new immunotherapeutic strategies. Cuproptosis patterns in COAD were shown to be linked with partial immune cell infiltration in this research, which examined the characterization of cuproptosis patterns based on CRGs. Principal component analysis (PCA) was employed to assess the genes linked to cuproptosis mortality. Finally, we compared the biological function and mutation differences between the groups, thus mining the potential chemotherapeutic drugs.

## 2. Results

### 2.1. Genetic and Transcriptional Alteration of CRGs in COAD

**Figure 1:**
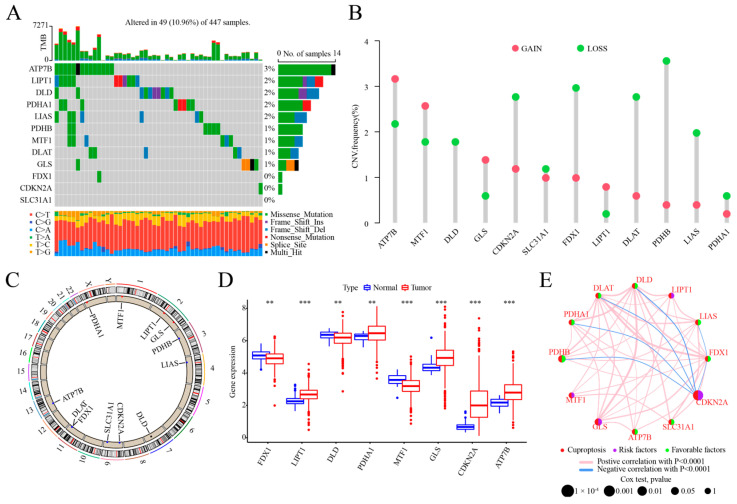
Genetic and transcriptional alteration of CRGs in COAD. (**A**) Mutation rates of 12 CRGs in 447 patients from the TCGA-COAD dataset; (**B**) frequency of CNVs involving gains and losses in CRGs; (**C**) chromosomal locations of CNV-altered CRGs; (**D**) differential expression of 8 CRGs in normal versus COAD tissues; significance levels are denoted as follows: ***, *p* < 0.001; **, *p* < 0.01; (**E**) interaction network diagram illustrating relationships among CRGs in COAD. Edges between genes indicate interactions among CRGs, with blue denoting negative correlations and pink indicating positive correlations. Line thickness indicates correlation strength. Circle size indicates the prognostic significance of CRGs, with *p*-values classified as *p* < 0.0001, *p* < 0.001, *p* < 0.01, *p* < 0.05, and *p* < 1. Purple circles denote risk factors, while green circles indicate protective factors.

In this study, 12 CRGs were analyzed for somatic mutation and CNV analysis. The results showed that among the 447 samples in the TCGA–COAD cohort, 49 samples (10.36%) had mutations in the CRGs (Figure 1A). However, the mutation frequency of each CRG was low (0–3%), not including FDX1, CDKN2A, and SLC31A1. Somatic CNV changes in the CRGs revealed the high amplification rates of ATP7B, MTF1, GLS, and LIPT1. However, CDKN2A, FDX1, DLAT, PDHB, LIAS, and PDHA1 were mainly copy number deletions, with similar amplification and deletion rates of SLC31A1, and DLD had only copy number deletions (Figure 1B). All 12 CRGs had CNV changes, and their altered chromosomal positions are shown by circle graphs (Figure 1C). We examined the mRNA expression levels in COAD and normal tissues, identifying significant differences in the expression of FDX1, LIPT1, DLD, DLAT, MTF1, GLS, CDKN2A, and ATP7B (Figure 1D). Combined with the CNV alteration results of the CRGs, we found that SLC31A1, DLAT, PDHB, and LIAS, with copy number deletions, showed no difference in expression between the normal samples and the COAD samples. In contrast to the normal samples, the COAD samples showed increased expression of ATP7B, GLS, and LIPT1, all of which had greater amplification rates, and decreased expression of MTF1. DLD and FDX1, with copy number deletions, had significantly lower expression levels. However, the expression levels of CDKN2A and PDHA1, with copy number deletions, were significantly increased. Thus, CNV may affect the mRNA expression of the CRGs, but is not the only major regulatory factor. We developed a network map depicting the interactions among the 12 CRGs (Figure 1E). The expression and survival data from the TCGA–COAD, GSE40967, and GSE17536 datasets were combined separately, removing the samples with less than 30 days of data for a total of 1159 samples. The prognostic importance of the 12 CRGs was highlighted through univariate Cox regression and the Kaplan–Meier analysis. The survival analysis results showed that (Supplementary Figure S1) eight CRGs, not including LIPT1, MTF1, SLC31A1, or ATP7B, were strongly associated with prognosis (*p* < 0.05). Patients with low CDKN2A and GLS expression, as well as those with high DLD, FDX1, DLAT, LIAS, PDHA1, and PDHB expression, exhibited an improved overall survival (OS), suggesting that the CRGs may impact the prognosis of COAD patients.

### 2.2. Identification of CRG Subtypes, Characterization, and Functional Enrichment of TME in COAD

This study employed consensus clustering analysis to categorize the 12 CRGs, utilizing cluster variables (k) from two to nine.

**Figure 2:**
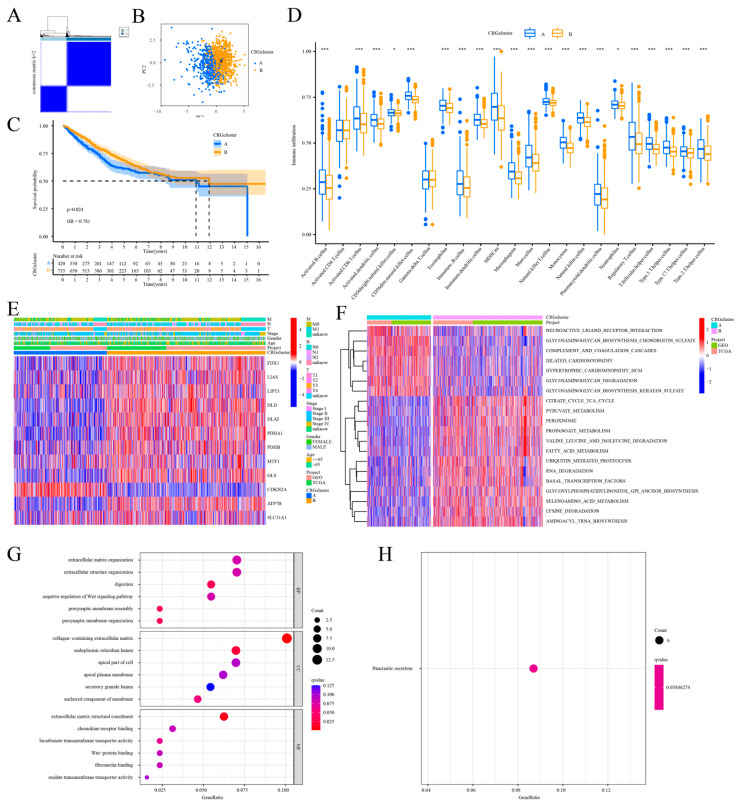
Characteristics of the CRGs in terms of subtype, clinicopathological factors, and biological aspects. (**A**) Consensus cluster analysis generated the consensus matrix diagram for the two related regions; (**B**) PCA emphasized the differences between the two subtypes; (**C**) OS rates were compared between the two subtypes; (**D**) evaluation of immune cell infiltration differences between subtypes; significance levels are denoted as follows: ***, *p* < 0.001; *, *p* < 0.05; and ns, not significant; (**E**) heatmap illustrating clinicopathological disparities and CRG expression levels across subtypes; (**F**) GSVA of biological pathways in subtypes, with red for activation and blue for inhibition; (**G**,**H**) GO and KEGG analyses of DEGs among CRG subtypes.

By the classification of K = 2, we obtained two clearly distinguished subclusters (A-n:447, B-n:764; Figure 2A). The two cuproptosis clusters were well-distinguished using principal component analysis (Figure 2B). The Kaplan–Meier survival curves for the COAD patients were generated for the two cuproptosis clusters. The patients in cuproptosis cluster B exhibited a higher overall survival (OS) rate (*p* = 0.024, Figure 2C). The analysis of immune cell abundance revealed that the patients in cuproptosis cluster A had a greater presence of immune-activated cells compared to those in cluster B. In the other 21 immune cell ratios, except activated CD4+ T cells and eosinophilia, the content was significantly different between both groups (*p* < 0.05, Figure 2D). Activated dendritic cells, natural killer (NK) cells, macrophages, mast cells, monocytes, and neutrophils were more abundant in cuproptosis cluster A. Simultaneously, we demonstrated the correlation between the two groups of cuproptosis and the clinicopathological characteristics using heatmaps. These characteristics mostly included age, gender, stage, and TNM (Figure 2E). The clinicopathological features were similar in both groups, according to the findings. The pathways associated with metabolic and cardiovascular illness (Figure 2F), such as dilated cardiomyopathy, glycosaminoglycan biosynthesis-keratan sulfate, and glycosaminoglycan degradation, were the most abundant in cuproptosis cluster A, based on the GSVA enrichment analysis. Cuproptosis cluster B predominantly involved metabolic pathways, including the TCA cycle, pyruvate, propanoate, fatty acid, and selenoamino acid metabolism. We identified 133 differentially expressed genes (DEGs) between the datasets, potentially offering insights into the molecular mechanisms underlying the cuproptosis pattern. Further GO and KEGG analyses of the GSVA enrichment findings revealed a significant enrichment of DEGs in immune function and stromal component-related terms, such as chemokine receptor binding and collagen-containing extracellular matrix (Figure 2G). The KEGG analysis indicated a significant enrichment of DEGs in the pancreatic secretion pathway (Figure 2H). These findings imply that the CRGs may be crucial in the immunoregulation and metabolism associated with the progression of COAD. 

### 2.3. Determination of Gene Subtypes Using DEGs

**Figure 3:**
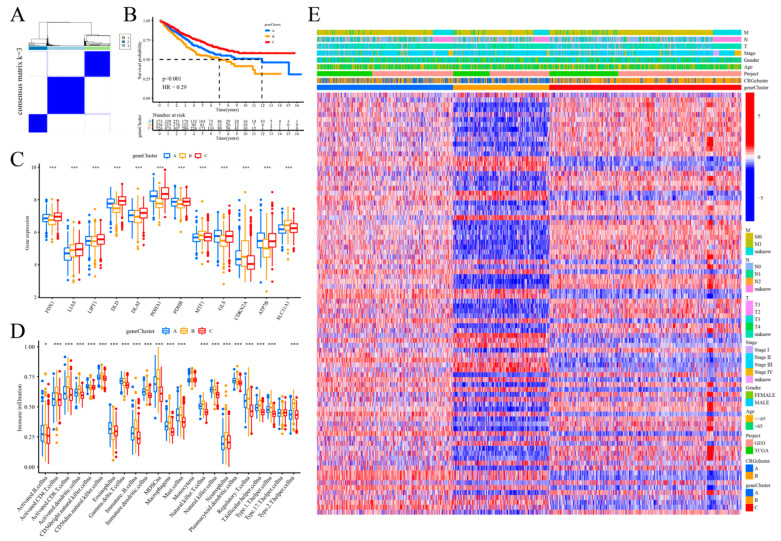
Gene subtypes were identified through differential expression analysis. (**A**) A consensus clustering matrix (k = 3) categorized COAD patients into three unique genomic subtypes; (**B**) the overall survival (OS) rates differed among these gene clusters; (**C**) CRG expression levels varied significantly across the clusters; (**D**) differences in immune cell infiltration levels across the three gene clusters; significance levels are denoted as follows: ***, *p* < 0.001; *, *p* < 0.05; (**E**) association between the three gene clusters and clinicopathological characteristics.

Following univariate Cox regression analysis, which demonstrated the predictive value of the 133 genes linked to the subtypes, 86 genes associated with prognosis were chosen for further examination of their molecular properties and prognostic significance. The patients were reclassified based on their prognostic genes using consensus cluster analysis. The analysis revealed that the samples were optimally clustered into three subtypes (k = 3), identified as gene cluster A (n = 386), gene cluster B (n = 275), and gene cluster C (n = 550) (Figure 3A). The analysis of overall survival (OS) among the three prognostic gene clusters revealed that gene cluster B had a significantly lower OS compared to the other two clusters (*p* < 0.001, Figure 3B). The expression levels of the 12 CRGs showed significant variation among the three gene clusters (Figure 3C). We observed a significant difference in the infiltration of partial immune cells between the three gene subtypes. Gene cluster B exhibited an increased presence of immune-active cells, including NK cells, macrophages, mast cells, neutrophils, activated dendritic cells, and activated B and T cells. Gene cluster A exhibited significantly higher levels in CD56 bright natural killer cells, eosinophils, and gamma delta T cells compared to other infiltration levels (Figure 3D). No significant differences in clinicopathological characteristics among the three categories were observed (Figure 3E).

### 2.4. Building and Testing the Cuproptosis Risk Score Model for Prognosis

**Figure 4:**
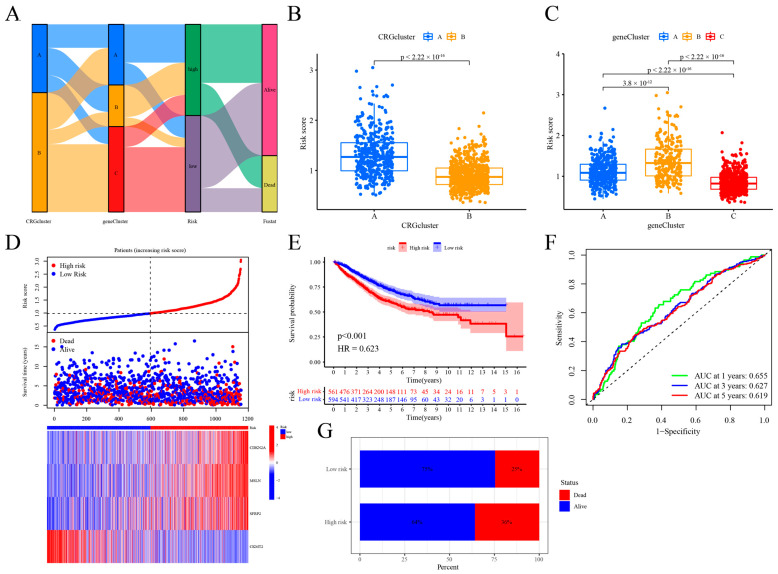
Development and validation of the cuproptosis risk score. (**A**) Sankey plots depict the relationships between cuproptosis clusters, gene clusters, cuproptosis risk scores, and survival status; (**B**) a comparison of cuproptosis risk scores across the two cuproptosis clusters; (**C**) analysis of cuproptosis risk score differences across three gene clusters; (**D**) examination of risk distribution, survival status, and related gene expression; (**E**) evaluation of overall survival (OS) rates between high-risk and low-risk groups; (**F**) ROC curve analysis for 1-, 3-, and 5-year survival predictions based on cuproptosis risk score; (**G**) comparison of survival rates between high-risk and low-risk patient groups.

This research used the DEGs linked to the cuproptosis subtype to generate a cuproptosis risk score. We show the relationships between gene clusters, cuproptosis risk scores, survival status, and clusters of cuproptosis (Figure 4A). With 577 patients in the training group and 578 in the test group, we used a 1:1 ratio. Using the Lasso Cox method on the DEGs linked with cuproptosis subtype provided the gene coefficients used to build the predictive features. We applied the Lasso Cox technique to the cuproptosis subtype-associated DEGs to derive the coefficients for the genes used in constructing the predictive features (Supplementary Figure S2A,B). The risk score was calculated based on four genes—MSLN, CDKN2A, SFRP2, and CKMT2—identified through multivariate Cox regression analysis (see Supplementary Table S1). The risk factors included CDKN2A, SFRP2, and MSLN, whereas the protective factor was CKMT2. The cuproptosis risk score was calculated by summing the products of specific coefficients and gene expressions: (0.1542 × CDKN2A), (0.0866 × MSLN), (0.0585 × SFRP2), and (−0.0983 × CKMT2). When we examined the two clusters of cuproptosis with the three clusters of genes, we found that cluster A had much higher risk ratings (*p* < 0.001, Figure 4B). Gene cluster B exhibited a significantly higher cuproptosis risk score compared to the other two clusters, with all three clusters showing statistically significant differences (*p* < 0.001, Figure 4C). There was a low-risk group of 561 patients whose cuproptosis risk scores were below the median and a high-risk group of 594 individuals whose scores were above the median. As the cuproptosis risk score rises, the distribution chart reveals a decline in survival time and an increase in mortality. There is a positive correlation between the cuproptosis risk score and the expression of the genes CDKN2A, SFRP2, and MSLN, while showing a negative correlation with the expression of gene CKMT2 (Figure 4D). The Kaplan–Meier survival curve confirmed that the patients in the low-risk group exhibited significantly better overall survival rates compared to the high-risk group (*p* < 0.001, Figure 4E). The high-risk group exhibited a 36% increase in mortality rate (Figure 4G). Aligned with previous OS findings for cuproptosis cluster A and gene cluster B, this indicates that a higher risk score correlates with a reduced OS rate. It illustrates the cuproptosis risk score’s area under the curve (AUC) as 0.655 at 1 year, 0.627 at 3 years, and 0.619 at 5 years of follow-up (Figure 4F). To evaluate the predictive power of the cuproptosis risk score, we calculated it for both the internal (training) and external (test) sets. Deaths in the test and training sets rose in tandem with increasing cuproptosis risk scores (Supplementary Figure S2C,D). A high-risk set was used for training, whereas a low-risk set was used for testing. In the survival analysis, the low-risk group in the training set showed superior outcomes compared to the test set (*p* = 0.004; *p* = 0.001). Supplementary This illustrates that the low-risk group exhibited a lower mortality rate compared to the high-risk group (25% vs. 35%; 24% vs. 38%; Supplementary Figure S2E,H; Supplementary Figure S2G,J). Cuproptosis risk scores maintain their high AUC values (Supplementary Figure S2F,I).

### 2.5. Clinical Classification and Significance of the Prognostic Model for Cuproptosis Risk Assessment

**Figure 5:**
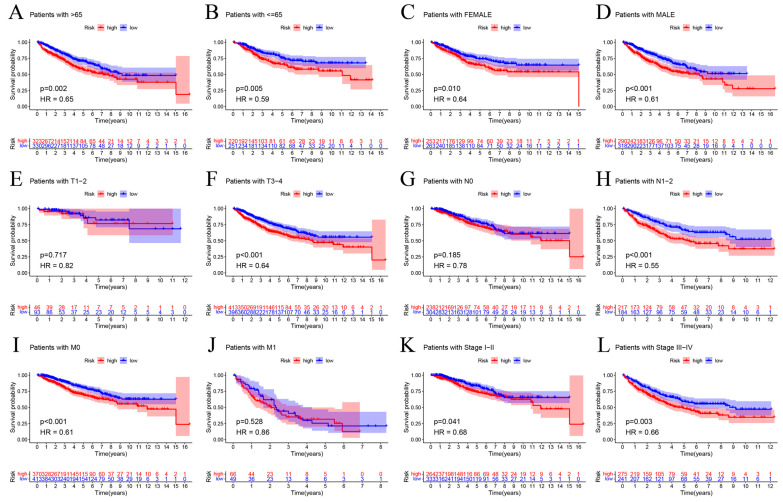
Correlation between cuproptosis risk score and clinicopathological subtype in COAD patients. (**A**,**B**) Age (≤65, >65); (**C**,**D**) gender (female, male); (**E**,**F**) T classification (T1–2, T3–4); (**G**,**H**) N classification (N0, N1–2); (**I**,**J**) M classification (M0, M1); (**K**,**L**) stage (I–II, III–IV).

We examined several clinicopathological variables (gender, age, stage, and TNM) to further confirm that the cuproptosis risk score was useful in predicting the clinical outcomes of the COAD patients. The subgroup analysis indicated that the patients in the low-risk group had a significantly better prognosis compared to those in the high-risk group (Figure 5A–L). This suggests that our cuproptosis risk score has a predictive value for different subgroups of clinicopathological characteristics.

**Figure 6:**
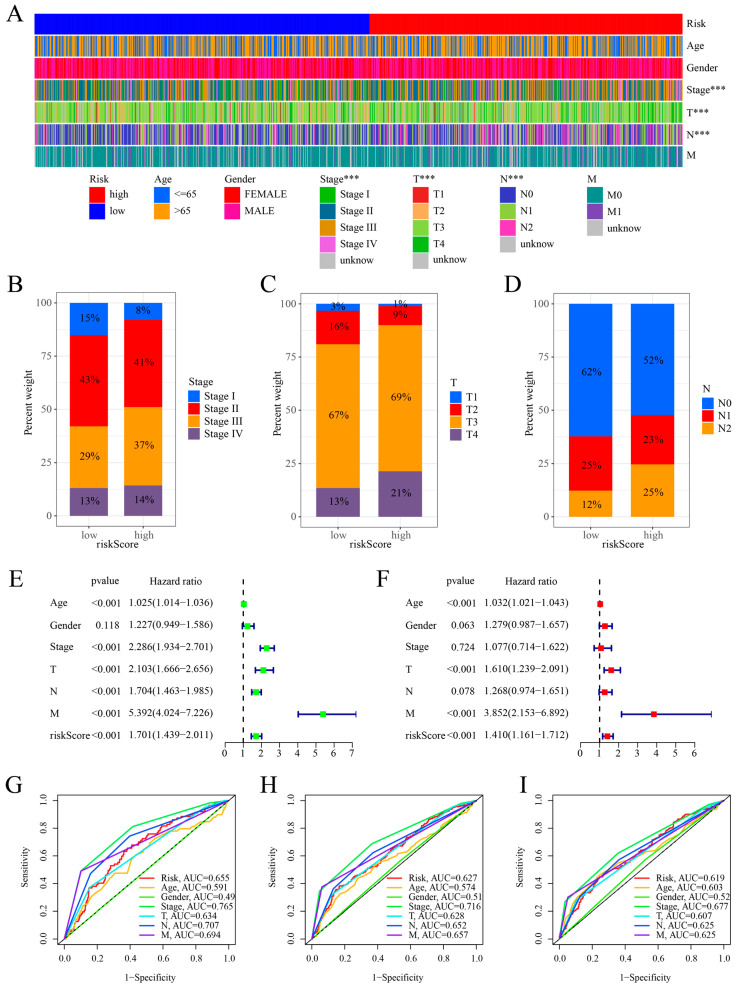
Clinical utility and independent prognostic assessment. (**A**) Heatmap showing the correlation between risk groups and clinicopathological characteristics; significance levels are denoted as follows: ***, *p* < 0.001; (**B**–**D**) proportions of risk groups across clinicopathological characteristics, including stage and T and N classifications; (**E**) univariate Cox regression analysis based on cuproptosis risk score and clinicopathological characteristics; (**F**) multivariate Cox regression analysis using the cuproptosis risk score and clinicopathological characteristics; (**G**–**I**) ROC curve assessing the risk model’s predictive capability at 1, 3, and 5 years.

We investigated the relationship between the cuproptosis risk score and various clinicopathological characteristics, finding significant differences in stage and TNM classifications. Notably, the patients in stage III, T4, N2, and M1 exhibited higher cuproptosis risk scores compared to other subgroups (Supplementary Figure S3). Additionally, the comparison of the two groups indicated significant differences in stage, T, and N related to the changes in clinicopathological characteristics (Figure 6A). The graphs indicate the percentages of people in the two groups (Figure 6B–D). The findings indicate that the low-risk group had fewer patients in advanced cancer stages and exhibited less clinical progression compared to those with palliative tumor lesions. This differed from the high-risk group. We used the cuproptosis risk score, age, gender, stage, TNM, and multivariate and univariate Cox regression approaches. The univariate Cox regression analysis demonstrated a significant correlation between the overall survival (OS) and factors such as cuproptosis risk score, age, stage, and T, N, M stages (Figure 6E). The multivariate Cox regression analysis confirmed a significant association between age, stage, T, N, M, and overall survival (OS). Our results also showed that the cuproptosis risk score was a reliable indicator of COAD prognosis (Figure 6F). The ROC curve demonstrates that our CRG prognostic risk model surpassed competitors in predicting 1-year, 3-year, and 5-year overall survival (OS) (Figure 6G–I). The cuproptosis risk score was validated as an independent predictor of COAD outcomes with a high predictive accuracy through ROC curve analysis of both univariate and multivariate Cox regression, alongside clinicopathological features, in training and test sets (Supplementary Figure S4).

### 2.6. Nomogram Construction and Validation

**Figure 7:**
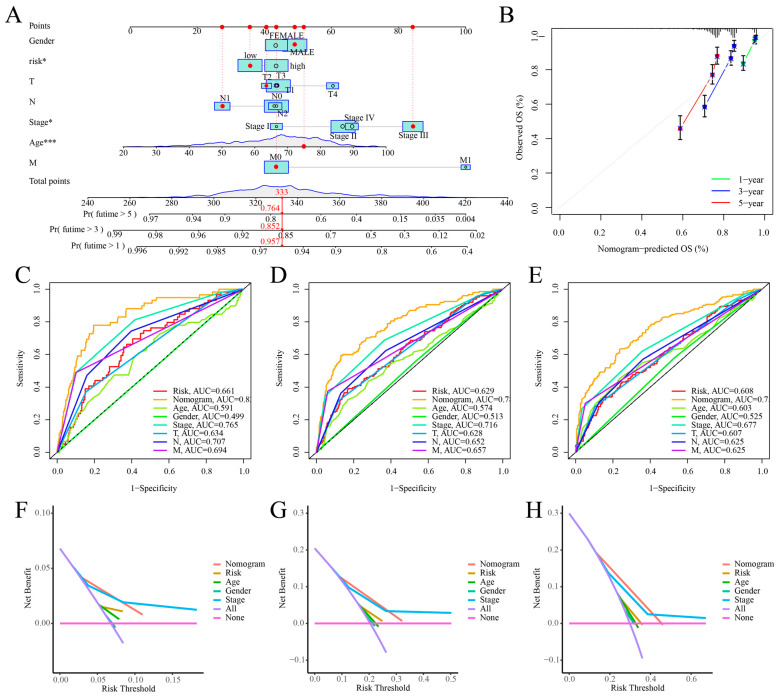
Development and validation of the nomogram. (**A**) Nomogram for predicting 1-, 3-, and 5-year overall survival (OS) in COAD patients; significance levels are denoted as follows: ***, *p* < 0.001; *, *p* < 0.05; (**B**) calibration curve of the nomogram; (**C**–**E**) ROC curves for 1-, 3-, and 5-year OS prediction; (**F**–**H**) DCA curves for 1-, 3-, and 5-year OS prediction.

In order to confirm that the cuproptosis risk score is useful for predicting the clinical outcome of COAD patients, this study developed a nomogram that combined the risk score with clinicopathological factors to predict outcomes for COAD patients (Figure 7A). It presents the calibration curve, confirming the reliability of our nomogram in accurately predicting 1-year, 3-year, and 5-year overall survival (OS) (Figure 7B). It also demonstrates that the nomogram and risk score effectively predicted 1-year, 3-year, and 5-year overall survival (OS) (Figure 7C–E). The DCA results indicated that the nomogram demonstrated superior accuracy in predicting the 1-year, 3-year, and 5-year survival rates of the COAD patients (Figure 7F–H).

### 2.7. Evaluation of the Immune Profile Based on Risk Factors

**Figure 8:**
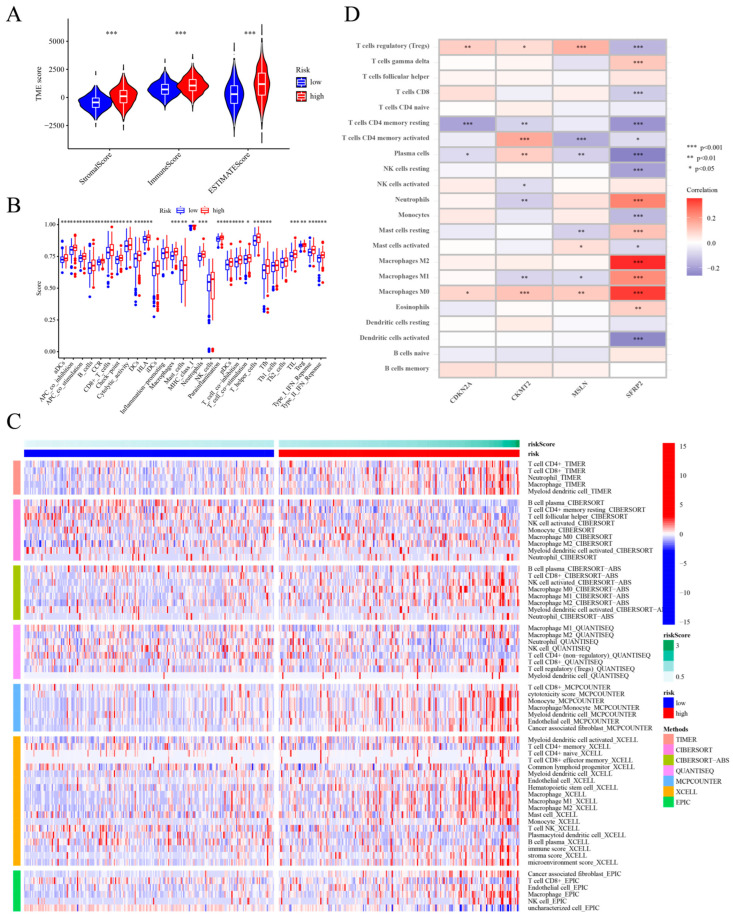
Examination of the immune landscape based on risk attributes. (**A**) The ESTIMATE algorithm to evaluate the association between cuproptosis risk groups and TME scores; (**B**) Analyzed immune cell abundance and immune function score disparities between high-risk and low-risk groups using ssGSEA; (**C**) Heatmap is provided to depict variations in immune cell content between these groups across different algorithms; (**D**) Correlation between immune cell abundance and the four genes in the constructed model is examined. Significance levels are denoted as follows: ***, *p* < 0.001; **, *p* < 0.01; *, *p* < 0.05.

Given the critical role of the tumor microenvironment (TME) in cancer progression and treatment, we set out to compare the immune systems of people at high and low cuproptosis risk. We employed the ESTIMATE program to analyze the TME scores, including stromal, immunological, and estimate scores, for various at-risk groups for cuproptosis. The high-risk group exhibited significantly elevated stromal, immune, and estimate scores compared to the low-risk group (*p* < 0.001, Figure 8A). We evaluated immune infiltration and function across the groups using the TIMER, CIBERSORT, quanTIseq, xCell, MCP-counter, and EPIC algorithms. The findings are shown as a heatmap (Figure 8C). The analysis revealed a significant difference in immune cell content between the high-risk and low-risk groups (*p* < 0.001; see Supplementary Table S2). Significant differences (*p* < 0.05) were observed in immune cell scores among the groups, specifically in dendritic cells (DCs), B cells, CD8+ T cells, activated dendritic cells (aDCs), macrophages, mast cells, neutrophils, plasmacytoid dendritic cells (pDCs), T helper cells, T follicular helper cells (Tfhs), tumor infiltrating lymphocytes (TILs), and regulatory T cells (Tregs). The higher risk group exhibited significantly elevated scores across 11 immune functions compared to the low-risk group (*p* < 0.05), including APC co-inhibition and co-stimulation, chemokine receptors, checkpoint, cytolytic activity, HLA, MHC class I, parainflammation, T cell co-inhibition and co-stimulation, and Type I and II IFN responses (Figure 8B). We continued by investigating the relationships between the cuproptosis risk score and the various immune cell subtypes. We discovered that 9 out of 22 cells relevant to the immune system had a significant association with the cuproptosis risk score. Among them, four types of immune cell infiltration (M0 macrophages, M1 macrophages, M2 macrophages, neutrophils) were positively correlated with the cuproptosis risk score (Supplementary Figure S5A–D). The cuproptosis risk score demonstrated an inverse correlation with five immune cell infiltrates: plasma cells, activated dendritic cells, resting NK cells, CD4 memory resting T cells, and activated T cells (Supplementary Figure S5E–I). We examined the association between the four genes used in the prognostic risk model and immune cell quantities, finding a strong correlation (Figure 8D), especially with SFRP2. In addition, SFRP2 had a strong positive correlation with both macrophages and neutrophils. The variables negatively correlated with SFRP2 comprised resting NK cells, plasma cells, CD4 memory resting T cells, CD8 T cells, Tregs, and activated dendritic cells. Based on these findings, the cuproptosis risk score may serve as a valuable clinical tool for managing COAD patients.

### 2.8. Association of Cuproptosis Risk Score with MSI, TMB, and CSC Index in COAD Patients

**Figure 9:**
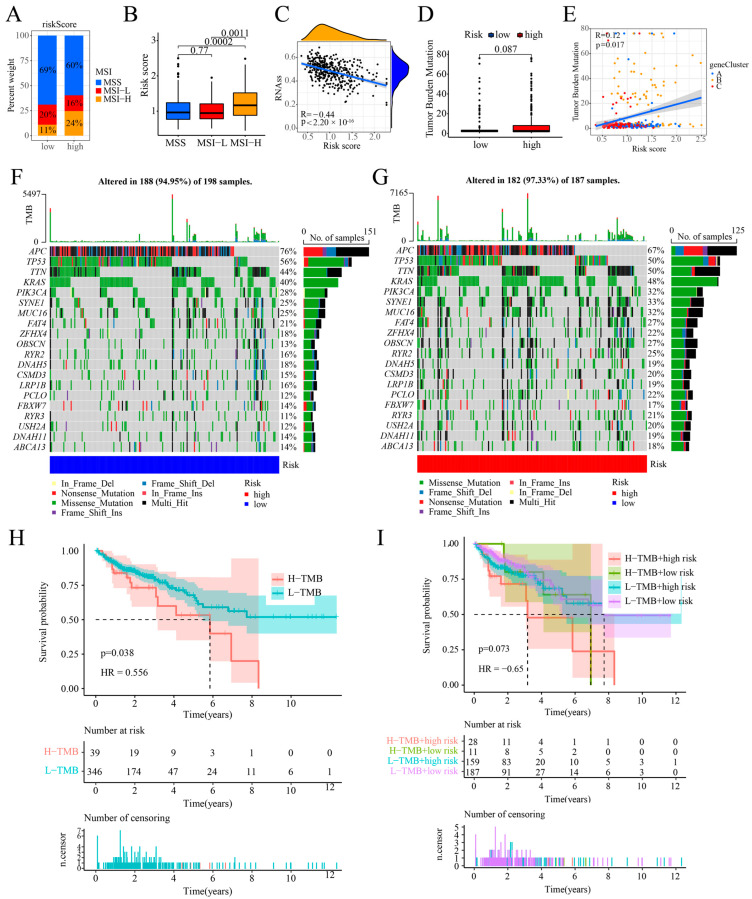
Relationship between cuproptosis risk score and MSI, TMB, and CSC index in COAD patients. (**A**,**B**) Correlation between cuproptosis risk scores and MSI; (**C**) relationship between cuproptosis risk scores and CSC index; (**D**) TMB across various risk groups; (**E**) Spearman’s correlation analysis of cuproptosis risk scores and TMB; (**F**,**G**) oncoplots of somatic mutation characteristics established with high and low cuproptosis risk scores; (**H**) prognostic analysis of TMB; (**I**) prognostic analysis between cuproptosis risk score and TMB.

Patients’ immune responses to immunotherapy may vary depending on their MSI, TMB, and CSC index. This study found that the low-risk group exhibited a higher proportion of microsatellite stability (MSS) and microsatellite instability-low (MSI-L) compared to the high-risk group. Microsatellite instability-high (MSI-H) was observed more frequently in the high-risk group (24%) compared to the low-risk group (11%; Figure 9A). The MSI-H group exhibited significantly higher cuproptosis risk ratings compared to the MSI-L and MSS groups (*p* < 0.001; Figure 9B). Prior research suggests that patients with MSI-H respond more effectively to immunotherapy, leading to improved outcomes [10]. Immunotherapy seems to be more effective for people with a high cuproptosis risk. The analysis revealed an inverse correlation between the cuproptosis risk score and the CSC index (R = −0.44, *p* <0.001; Figure 9C). This indicates that the COAD cells with lower cuproptosis risk scores exhibited more prominent stem cell characteristics and a reduced level of cell differentiation. We examined the TMB differences between high and low cuproptosis risk groups, finding no significant variation (*p* = 0.087; Figure 9D). The analysis demonstrated a significant positive correlation between the cuproptosis risk score and the TMB across the three gene clusters (*p* < 0.017; Figure 9E). Both the high-risk (Figure 9F) and low-risk (Figure 9G) groups’ top 20 mutant genes are shown via the oncoplots. The top ten mutations identified in both the high-risk and low-risk categories were APC, TP53, TTN, KRAS, SYNE1, PIK3CA, MUC16, FAT4, ZFHX4, and OBSCN. Although there was no statistically significant difference, APC and TP53 mutation frequencies were higher in the low-risk group (76%) compared to the high-risk group (56%). However, elevated mutation levels have been noted in other genes within high-risk groups. Given the clinical significance of TMB, we categorized the COAD patients into high and low TMB groups based on mutation frequency and conducted a survival prognostic analysis. The patients in the group with lower TMB had improved OS rates in comparison to the group with greater TMB levels (*p* = 0.038; Figure 9H). We concurrently conducted a subgroup survival analysis incorporating both the TMB and the risk score. The OS rate was lower in the high-risk, high-TMB group than in other groups, but this difference was not statistically significant (*p* = 0.073; Figure 9I).

### 2.9. Accuracy and Significance of Modelling for Prediction of Immunotherapy and Drug Sensitivity

**Figure 10:**
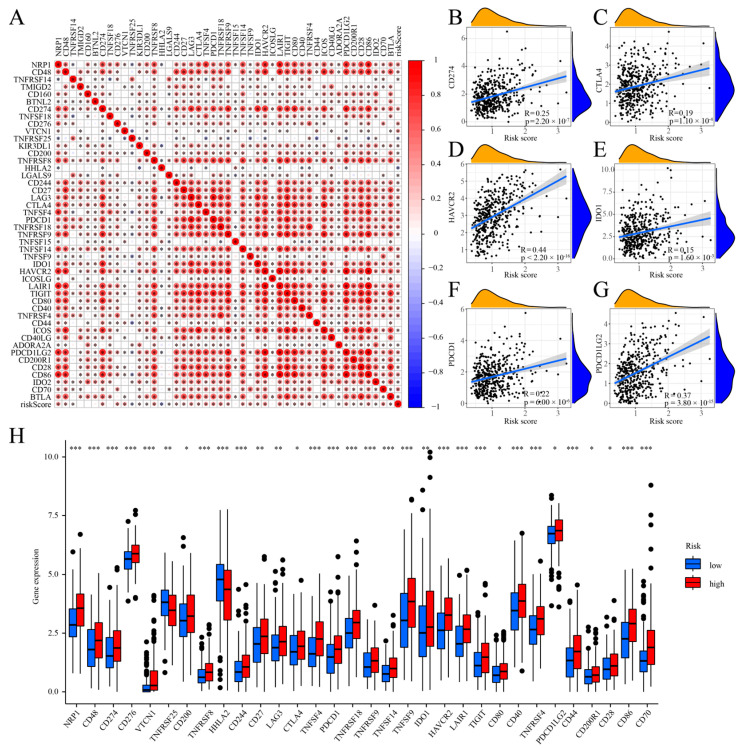
Examination of the relationship between prognostic features derived from cuproptosis risk scores and immune checkpoint genes. (**A**) Relationship between cuproptosis risk scores and immune checkpoints; (**B**–**G**) Correlation between the cuproptosis risk scores and the 6 key immune check-point genes (CD274, CTLA-4, HAVCR2, IDO1, PDCD1, and PDCD1LG2); (**H**) Differential ex-pression of immune checkpoint genes between high- and low-risk groups. Significance levels are denoted as follows: ***, *p* < 0.001; **, *p* < 0.01; *, *p* < 0.05.

**Figure 11:**
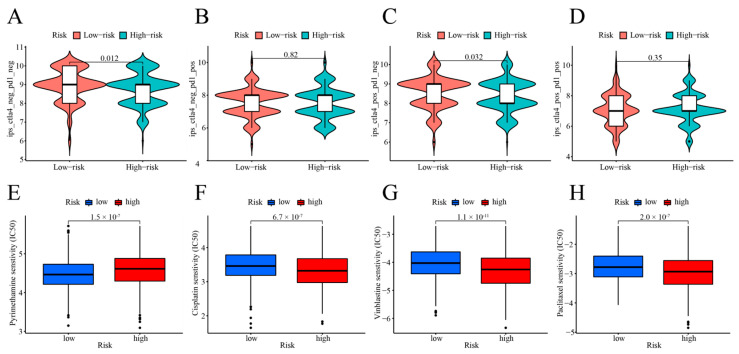
Analysis of immunotherapy and drug sensitivity in IPS. (**A**) No use of PD-1 or CTLA-4 inhibitors; (**B**) use of a CTLA-4 inhibitor alone; (**C**) use of a PD-1 inhibitor alone; (**D**) combined use of CTLA-4 and PD-1 inhibitors; (**E**–**H**) association between cuproptosis risk scores and drug sensitivity.

One of the biomarkers for selecting for immunotherapy in COAD patients is immune checkpoint expression, and we first investigated the correlation between the cuproptosis risk score and the 47 immune checkpoint genes. A statistically significant correlation was observed between the cuproptosis risk score and the expression of 36 immunological checkpoints (*p* < 0.05; Figure 10A). Additionally, significant positive correlations were observed with six key immunological checkpoint genes: CD274, CTLA-4, HAVCR2, IDO1, PDCD1, and PDCD1LG2 (*p* < 0.001; Figure 10B–G). We analyzed immune checkpoint gene expression differences between high- and low-risk groups, finding significant variations in 32 of 47 genes (*p* < 0.05; Figure 10H). The low-risk group exhibited significantly higher levels of the immune checkpoint proteins HHLA2 and TNFRSF25 compared to the high-risk group (*p* < 0.01; *p* < 0.001). The low-risk group showed a substantially reduced expression of the other 30 immunological checkpoints when contrasted with the high-risk group. This was especially evident for the six key genes associated with immune checkpoint pathways: CD274, CTLA-4, HAVCR2, IDO1, PDCD1, and PDCD1LG2. This provides further evidence that the associated immune checkpoint inhibitors (ICIs) may have a more beneficial effect on people at high risk. The cuproptosis risk score model effectively stratified the COAD patients, aiding in the selection of appropriate ICIs. We utilized IPS data from the COAD patients in the TCIA database to categorize them into high- and low-risk groups based on their immunotherapy response. The IPS plot results indicated that the low-risk individuals experienced greater effects from immunotherapy compared to high-risk patients, both without PD-1 and CTLA-4 inhibitors (*p* = 0.012) and with CTLA-4 inhibitors alone (*p* = 0.032; Figure 11A,B). It illustrates our investigation into the effectiveness of targeted therapies and commonly used chemotherapeutic drugs concerning cuproptosis risk ratings in COAD patients (Figure 11E–H), alongside ICI treatment, rather than solely using PD-1 inhibitors (*p* = 0.82; Figure 11C) or in combination with CTLA-4 (*p* = 0.35; Figure 11D). The results indicate that the low-risk patient group demonstrated significantly lower IC50 values for pyrimethamine (*p* < 0.001; Figure 11E), vorinostat (*p* < 0.001; Supplementary Figure S6A), and methotrexate (*p* < 0.001; Supplementary Figure S6B). The IC50 values for Cisplatin, Vinblastine, Paclitaxel (*p* < 0.001; Figure 11 F–H), and additional chemotherapeutic and targeted drugs, were significantly reduced in the high-risk group (*p* < 0.001; Supplementary Figure S6C–L). This provides further evidence that high-risk individuals may benefit from chemotherapy and tailored therapeutic regimens.

### 2.10. Experimental Validation of Critical Gene Expression Levels Using qPCR and IHC

**Figure 12:**
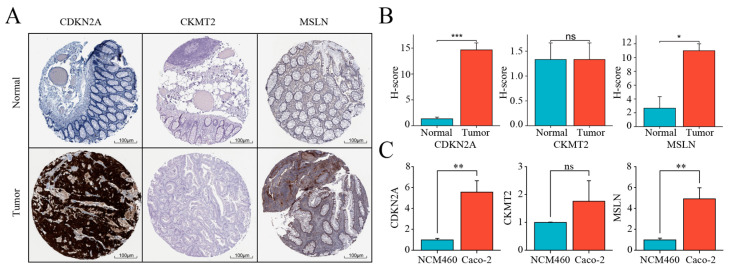
Biological validation results of key genes CDKN2A, CKMT2 and MSLN. (**A**) Differences in three convenient expressions of CDKN2A, CKMT2 and MSLN between human normal tissues and tumor tissues obtained based on IHC from HPA database; (**B**) H-score of IHCs; (**C**) results of RT-qPCR to detect the differential expression of transcript levels of CDKN2A, CKMT2, and MSLN in normal and tumor cell lines. Significance levels are denoted as follows: ***, *p* < 0.001; **, *p* < 0.01; *, *p* < 0.05; and ns, not significant.

Firstly, using the relevant data of IHC in the HPA database, the key genes CDKN2A, MSLN, and CKMT2 were compared at the protein level, and it was found that CDKN2A and MSLN were significantly highly expressed in the tumor tissues (*p* < 0.001; *p* < 0.05; Figure 12A,B), which was consistent with our prediction. Meanwhile, through the RT-qPCR experiments, we also obtained consistent results (*p* < 0.005; Figure 12C). This is enough to suggest that the key genes screened in this paper have important significance in the process of tumorigenesis and development.

## 3. Discussion

COAD is a common gastrointestinal tumor, but the factors affecting its development and metastasis are not fully understood. Recent research has identified cuproptosis as a novel form of cell death. Copper ionophores alter intracellular metabolite levels, ultimately causing cell death, indicating their potential as a novel treatment strategy for cancer cells with these metabolic traits. Research indicates that CRG signatures can serve as prognostic indicators, influencing the progression of clear cell renal cell carcinoma (ccRCC) [11]. However, the role of CRGs in COAD has not been reported.

In this study, we first illustrated the somatic mutations, CNV frequency, and chromosomal location in COAD by extracting 12 CRGs, combined with survival data, to conduct a preliminary analysis of the prognosis of the CRGs, and we constructed a prognostic network map. We combined the survival data with the results of the consensus clustering analysis of the 12 CRGs. The patients in cuproptosis cluster group B exhibited a significantly better overall survival prognosis compared to those in cluster group A. This finding indicates the presence of two distinct groups of molecular subtypes, with significant differences in their molecular profiles that impact patient prognosis. Building on this, we further investigated the immune infiltration and functional enrichment associated with these two groups. Our findings indicate that the patients in cuproptosis cluster A showed increased immune cell infiltration, implying a strong association between cuproptosis and immune infiltration. Secondly, the GSVA results for the individuals in cuproptosis cluster A showed significant enrichment in metabolism-related pathways, especially the TCA cycle. Copper ion build up in cells and direct binding to the lipoylated components of TCA constitute the major step of cuproptosis. Multiple mechanisms may interact to enhance COAD cell proliferation, migration, and invasion while reducing the therapeutic impact [12]. One of these aspects is the metabolic interaction between tumor cells, TME, and intestinal microbiota. We subsequently identified three gene clusters by examining the differentially expressed genes (DEGs) between the two cup-shaped drop clusters. Our refined classification of CRG molecular subtypes revealed notable survival rate differences among the three gene clusters. This indicates that the CRGs could be used as predictors for clinical outcomes and the efficacy of immunotherapy. We developed a cup-shaped drop risk scoring system and a prognostic model based on four CRGs. CDKN2A is crucial for cell cycle regulation, inhibiting cell proliferation, promoting apoptosis, and serving as a biomarker for invasive meningioma. The cell surface glycoprotein encoded by MSLN is closely associated with the development of pancreatic and ovarian cancers, and it serves as a target antigen for CAR-T therapy [13,14]. SFRP2, a secreted protein, inhibits the WNT signaling pathway. Its downregulation abnormally activates this pathway, facilitating tumor progression [15,16]. CKMT2, a mitochondrial creatine kinase, regulates mitochondrial function and is considered a potential biomarker [17,18]. Cuproptosis-related genes are crucial in tumor initiation and progression, underscoring their potential as clinical biomarkers. Moving forward, we will explore the clinical applications and prospects of these genes in greater depth [19,20]. The model was evaluated on both training and test sets to verify its accuracy. The patients with a reduced cuproptosis risk demonstrated better overall survival, prognosis, and prognostic prediction accuracy at 1, 3, and 5 years. The patients with certain subtypes of clinicopathological characteristics had their OS rates impacted by the cuproptosis risk score, according to clinical value analysis and clinical categorization of the cuproptosis risk score prognostic model. Independent prognostic research using both univariate and multivariate variables confirmed that the cuproptosis risk score is a useful and accurate predictor of COAD prognosis. Improving the cuproptosis risk score’s prediction capacity, we then created a nomogram by integrating the cuproptosis risk score with clinicopathological parameters. This result further validates the prognostic model we constructed, suggesting that it can serve as a prospective predictive tool in clinical diagnosis. This model can improve patient treatment outcomes by allowing for more accurate risk stratification in clinical practice.

Cuproptosis is a newly identified form of cell death impacting disrupted cellular metabolism. Our study indicates that CRGs are significantly enriched in metabolic pathways, influencing the tumor immune microenvironment and promoting tumor development. We analyzed the immune landscape according to cuproptosis risk features to investigate the CRGs’ role in the tumor immune microenvironment. We started by using the estimation program to compare the immunological, stromal, and estimation scores for the TME in the groups. The TME comprises two primary cell types: immune cells and stromal cells. The degree to which these cells infiltrate both the tumor tissue and the TME cells greatly affects the prognosis [21]. The estimation approach suggests that the prognosis can be predicted using both the immunological and stromal scores. The higher the stromal score and the immunological score, the less pure the tumor is, which makes it more favorable for tumor genesis and development. This study revealed that stromal, immune, and estimation scores were significantly elevated in the high-risk group compared to the low-risk group. The risk score had a negative association with the tumor purity but a positive correlation with the stromal, immunological, and estimation scores. A high-risk score may enhance tumor development and progression, adversely affecting the patient prognosis. The overall survival analysis indicates that the patients in the high-risk group had a comparatively shorter survival time. The prognostic outcome of the tumor patients may be related to the extent of tumor immune cell infiltration, and it has been shown that tumor immune infiltration is more helpful in predicting the recurrence of COAD disease compared with other transcriptome-based biomarkers [22]. The ssGSEA analysis indicated significantly elevated immune cell scores, including immunosuppressive cells like macrophages, mast cells, neutrophils, and Tregs, in the high-risk group compared to the low-risk group. Tumor-associated macrophages (TAMs) in the tumor stroma can recruit Tregs by secreting cytokines and chemokines. Furthermore, TAMs can promote the proliferation, migration, and angiogenesis of tumor cells, inhibiting the anti-tumor immune response of T cells and interrupting the interaction of immune cells, thus forming the immunosuppressive microenvironment of COAD [23]. The TAM mainly presents the phenotype of the M2-like, immunosuppressive macrophages in the TME [24]. Furthermore, the TAM correlates with an unfavorable tumor prognosis. Perivascular mast cells facilitate angiogenesis and tumor progression in both early and late stages of COAD, with their abundance linked to a poor prognosis in COAD patients [25,26]. Apoptotic tumor cells release chemokines that attract neutrophils to the tumor, which interact with immediately adjacent macrophages to promote the formation of an immunosuppressive TME, and this can lead to tumor recurrence after treatment with chemotherapeutic agents [27]. However, the intratumor increase in neutrophils was associated with a malignant phenotype, helping to predict the poor prognosis of COAD [28]. Research indicates that Treg infiltration serves as an independent prognostic risk factor in COAD, correlating with reduced disease-free survival (DFS) and overall survival (OS) in CRC patients with elevated invasive Tregs [29]. Cuproptosis can protect tumors from the body’s natural anti-tumor immune response by creating an immunosuppressive microenvironment. This, in turn, causes the immune system to evade detection, which in turn encourages the tumors to grow and migrate even further. Consequently, the risk of the patient worsening and dying is heightened, and the reason for the high-risk group’s poor prognosis may be revealed. The activation of both Type I and Type II IFN responses in the high-risk immunosuppressed patients indicates a potential positive response to immunotherapy [30,31]. A negative correlation was observed between the cuproptosis risk score and the levels of five types of immunoactivated cells in the blood: plasma cells, activated dendritic cells, resting NK cells, CD4 memory T cells, and CD4 memory resting T cells. Thus, the immunological competence declines as the cuproptosis risk scores rise, hastening the invasion, migration, and proliferation of COAD.

Immunotherapy, as the most cutting-edge cancer treatment program, can more accurately predict the effect of immunotherapy through the biomarkers of four classes of immunotherapy, including PD-L1 expression, TMB, MSI detection, and TIL detection. The application and promotion of anti-PD-L1 antibody serves as a representative ICI; it provides a new therapeutic route for most tumor patients [32,33]. A prior study indicated that patients with MSI-H tumors in COAD exhibited a significantly greater sensitivity and benefit from ICI treatment compared to those with MSS/MSI-L tumors [34]. The study found a significant association between the low-risk group and MSS status, as well as between the high-risk group and MSI-H status. Despite the poorer prognosis in the higher risk group, new strategies may enhance the treatment outcomes and overall survival in this cohort. CSCs are the basis of tumor recurrence, metastasis, and drug resistance [35], and they have the ability of self-renewal. Due to its resistance to most therapies, including chemotherapy and radiotherapy, it has become a major resistance in the process of cancer treatment [36]. Targeting cancer stem cells (CSCs) through immune-based approaches plays a crucial role in cancer treatment [37]. Our research identified an inverse relationship between the cuproptosis risk score and the CSC index, suggesting that the low-risk group may exhibit reduced chemosensitivity and tumor growth.

A high TMB may be a reliable biomarker for forecasting the tumor response to ICIs, such as COAD, according to related research, which has shown that the MSI status substantially raises the TMB [38]. Although no statistically significant differences in the TMB were observed between the high- and low-risk groups, a positive correlation was identified between the TMB and the cuproptosis risk score, indicating that the TMB may serve as a measure of immunotherapy efficacy. We observed that low TMB patients with COAD had a significantly better OS rate compared to high TMB patients. Subtyping the TMB patients according to their risk ratings did not reveal any statistically significant differences. Research has shown that immune checkpoint molecule expression is predictive of immunotherapy efficacy and that it plays a significant role in tumor immune escape [38]. Cancer of the immune system (COAD) patients have a reason to hope for a cure with targeted immune checkpoint blockade (ICB) therapy [39]. Their effects depend on tumor immune infiltration, immune checkpoint expression, TMB, and neoantigen formation [40]. One of the most important and successful ICB therapies at the moment is treatment with PD-1 inhibitors; another is CTLA-4 inhibitors [41]. This study found a positive correlation between the cuproptosis risk score and the six key ICB targets. The expression levels of these genes were significantly higher in the high-risk cuproptosis group compared to the low-risk group. The tumor cells in the high-risk group may express immune checkpoint molecules to evade immune attacks. PD-1/PD-L1 antibody treatment may be clinically effective for high-risk patients receiving corresponding immune checkpoint therapy. The IPS results indicated that the COAD patients in the low-risk group were more likely to benefit from immunotherapy, regardless of whether it was administered without PD-1 and CTLA-4 inhibitors or with only CTLA-4 inhibitors, compared to the high-risk patients. However, there was no distinction between the patients treated solely with CTLA-4 inhibitors and those receiving a combination of PD-1 and CTLA-4 inhibitors. This risk scoring model improves the prediction of the COAD patients’ responses to immune checkpoint inhibitors, enabling more personalized treatment strategies in clinical plans. This approach can effectively improve patient outcomes and provides a critical foundation for future clinical trials.

In both the high- and low-risk COAD patients, we examined the IC50 values of several anticancer drugs. Less sensitive to Cisplatin, Vinblastine, Paclitaxel, and most targeted and chemotherapeutic medications were the low-risk patients, whereas Pyrimethamine, Vorinostat, and Methotrexate was less sensitive in the high-risk patients. Treatment resistance may be effectively addressed by using cuproptosis risk scores as prognostic markers in the COAD patients prior to chemotherapy, while subtyping the chemotherapeutic drugs aids in the treatment selection.

While we have included numerous public datasets as validation sets for the prediction model, clinical prospective studies remain essential. The integration of real-world data can further validate the accuracy of the prognostic model. Although we propose potential drug treatments based on this model, we cannot predict the safety and effectiveness of these drugs in actual individual cases. Clinical trials are crucial for revealing both the therapeutic benefits and potential risks of drug treatments for patients, making them necessary for confirming the results of bioinformatics predictions.

In summary, no studies have specifically investigated the relationship between CRGs and COAD. This study innovatively combined genetic subtype analysis, tumor mutation characteristics, and immune landscape assessment to offer a comprehensive approach for prognostic evaluation. We not only examined the impact of CRGs on the patients’ overall survival but also enhanced the model’s stability and predictive ability through internal and external validation. Additionally, our in-depth exploration of potential drug targets offers new insights for the clinical treatment of colorectal cancer. By combining CRGs with drug sensitivity data, we can predict beneficial drugs for patients, thereby aiding in the development of individualized treatment strategies. These findings pave the way for new approaches in risk prediction and the precision treatment of colorectal cancer. 

## 4. Materials and Methods

### 4.1. Multi-Omics Data Sources and Processing

To gather transcriptomic and clinical data for patients with COAD, the researchers accessed two databases: the Cancer Genome Atlas (TCGA) (https://www.cancer.gov/, accessed on 20 October 2024) and the Gene Expression Omnibus (GEO) (https://www.ncbi.nlm.nih.gov/geo/, accessed on 20 October 2024). The TCGA–COAD dataset comprised 480 samples from COAD patients and 41 from normal tissues. Genomic analysis (FPKM), clinical data, somatic mutations, copy number variation (CNV), and both tumor and normal tissue samples were collected from patients up to 8 May 2022. We converted the TCGA–FPKM COAD data to transcripts per million (TPM) to enable comparison with microarray data. The GEO data came from two sources: the GSE17536 dataset (177 samples) [42] and the GPL570 Expression Spectrum-chip GSE40967 dataset (585 samples) [43]. Using the ‘ComBat’ method from the R package ‘sva’ to integrate and correct batch effects in the expression data across three datasets resulted in 1211 samples available for further analysis. The 12 CRGs were extracted from the literature [44].

### 4.2. Multi-Omic Landscape Analysis of the CRGs in COAD

The ‘maftools’ package (https://www.bioconductor.org/packages/release/bioc/html/maftools.html; accessed on 20 October 2024) was utilized to assess, summarize, and visualize the CRG mutation data and tumor mutation burden (TMB) values for the CRC samples, including the generation of oncoplots. The CNV frequency of the CRGs was also assessed. The ‘limma’ and ‘ggpubr’ packages were employed to analyze CRG expression in the TCGA–COAD dataset, facilitating the comparison of the 12 CRGs between normal and tumor tissues and identifying significantly different genes. Gene expression data from TCGA–COAD and GEO were integrated with clinical survival information to assess CRG expression levels. Univariate Cox regression analysis was conducted using the ‘survival’ and ‘survminer’ packages to evaluate the prognostic significance of CRGs. Prognostic interaction network plots for the CRGs were generated using the ‘igraph’ and ‘reshape2’ packages, applying the criteria |Pearson R| > 0.2 and *p* < 0.05. 

### 4.3. Analysis of Consensus Clusters, Functional Biological Enrichment, and Immune Cell Infiltration Traits in Cuproptosis Clusters

CRG expression profiles were used to classify COAD patients, who were then subjected to a consensus clustering analysis to look for different signs of cuproptosis. Regarding consensus unsupervised clustering analysis, this research made use of the ‘ConsesusClusterPlus’ program (https://bioconductor.org/packages/release/bioc/html/ConsensusClusterPlus.html; accessed on 20 October 2024) [45]. PCA was employed to identify the two cuproptosis clusters among all COAD patients utilizing the ‘ggplot2’ package. Survival was compared between the different cuproptosis clusters using the Kaplan–Meier analysis. Heat maps of clinical factors were used to visualize the relationship between cuproptosis clusters using the Pearson chi-square test. The ‘GSVA’ program utilizes the marker gene set (c2.cp.kegg.v7.2) for Gene Set Variation Analysis (GSVA) to explore biological variations across different populations [46]. We utilized single-sample gene set enrichment analysis (ssGSEA) on 23 gene sets associated with immune cells [47].

### 4.4. Identification of Differentially Expressed Genes (DEGs) and Functional Enrichment Analysis of Cuproptosis Clusters

Differentially expressed genes (DEGs) were identified between the two cuproptosis clusters using the ‘limma’ and ‘VennDiagram’ packages, applying a logFC filter of 0.585 and a corrected *p*-value threshold of *p* < 0.05 [48]. Gene Ontology (GO) and Kyoto Encyclopedia of Genes and Genomes (KEGG) analyses were conducted on the DEGs to identify pathways linking enriched GO terms with cuproptosis clusters [49].

### 4.5. Construction of a Prognostic Model for CRGs

To find genes related to COAD prognosis, univariate regression was used. Patients were classified into subtype groups A, B, and C based on unsupervised clustering of prognostic CRG expression related to cuproptosis genes. This study’s 577 COAD patients were randomly assigned to either the training or test group. We employed multivariate Cox regression, the ‘glmnet’ program, and Lasso Cox regression to refine the selection of candidate genes for cuproptosis prognosis. The last step was to build a prognosis model using the cuproptosis risk score. The risk score was calculated using the formula risk score = Σ(EXPI × coefi), where EXPI represents the expression level and risk coefficient for each gene. The overall sample, as well as the training and test groups, was categorized into low and high risk based on the median risk score. ROC and Kaplan–Meier survival analyses were conducted.

### 4.6. Identification of the Clinical Utility Value of the Risk Score

Clusters of genes, cuproptosis risk scores, clusters of cuproptosis, and survival statuses are shown in Sankey plots for individual patients. The WilcoxTest evaluates prognostic gene clusters or clusters of cuproptosis patients by comparing their risk scores. Further analysis examined the correlation between the cuproptosis risk score and clinical variables including age, gender, clinical stage, and TNM. Independent prognostic analysis of risk scores was used in the training and test sets. We performed stratified analyses to compare the accuracy of cuproptosis risk ratings in predicting age, gender, clinical stage, and TNM subgroups.

### 4.7. Development and Validation of a Nomogram

Using the ‘rms’ package, predictive nomograms were developed based on clinical characteristics and cuproptosis risk scores derived from the independent prognostic analysis findings [50]. Nomograms were evaluated using time-dependent ROC curves for survival at 1, 3, and 5 years. Calibration plots showed the concordance between predicted and observed survival events at 1, 3, and 5 years. Decision curve analysis (DCA) was performed with the R package ‘ggDCA’ to improve the assessment of the nomogram’s prognostic accuracy.

### 4.8. Immunolandscape Analysis

A thorough analysis of immune infiltration and function was achieved by integrating results from multiple algorithms. For the purpose of forecasting immune infiltration and stroma state in the TME, the ‘ESTIMATE’ algorithm may be used [51]. The program evaluates the tumor’s purity, stroma composition, and immune cell concentration. Using the ‘ggpubr’ and ‘reshape2’ packages, we compared the dangers to the TME caused by low and high cuproptosis. A link between immune cell counts and risk scores was extracted by cycling all immune cells using the ‘tidyverse’ and ‘ggExtra’ packages. The percentage of 22 distinct categories of immune-related cell subtypes was shown using the CIBERSORT algorithm [52]. The use of ssGSEA to categorize immune cells and immunological activity into high- and low-risk groups for cuproptosis aimed to investigate immune modulatory pathways. The TCGA file was used to estimate the quantity of immune cells infiltrating a sample. This dataset was obtained from the TIMER2.0 database [53]. The analysis results were mapped using TIMER [54], CIBERSORT [55], quanTIseq [56], xCell [57], MCP-counter [58], and EPIC [59].

### 4.9. Association of COAD Cuproptosis Risk Scores with TMB, Microsatellite Instability (MSI), and Cancer Stem Cell (CSC) Index

Using the ‘maftools’ program, we analyzed the mutation rates of genes in two groups: high and low cuproptosis risk. Oncoplots were used to display the top 20 genes ranked by mutation frequency. The correlation between CRG predictive genotyping and TMB differences in high and low cuproptosis risk groups was analyzed using the ‘limma’ and ‘ggpubr’ programs. The appropriate TMB differentiation cutoff, as well as the TMB survival curve and survival curves combining high and low cuproptosis risk, was produced using the ‘survival’ and ‘survminer’ packages. We also examined the relationship between the cuproptosis risk score and both the MSI and CSC indexes [60].

### 4.10. Analysis of Immunotherapy and Drug Sensitivity

Current cancer treatments encompass chemoradiotherapy, targeted therapy, and immunotherapy, with immune checkpoint expression being pivotal to immunotherapy’s success. We analyzed the relationship between the cuproptosis risk groups and 47 immune checkpoint genes to clarify the gene signatures’ role in COAD immunotherapy and identify more effective treatments for high- and low-risk patients. The TCIA database (https://tcia.at/home; accessed on 8 May 2022) offers the immunophenoscore (IPS) for COAD patients, enabling comparisons of immunotherapy efficacy among groups with varying cuproptosis risk. We utilized the ‘pRRophetic’ package to estimate the half maximal inhibitory concentration (IC50) for both high and low cuproptosis risk groups, aiming to assess variations in chemotherapeutic drug efficacy between these groups [61].

### 4.11. Cell Culture

Caco-2 and NCM460 cells were supplied by the Cell Resource Center at the Institute of Basic Medical Sciences, CAMS/PUMC, Chinese Academy of Medical Sciences (Beijing, China). In vitro studies involved culturing NCM460 cells in DMEM with 10% fetal bovine serum and 1% penicillin/streptomycin, and Caco-2 cells in DMEM with 20% fetal bovine serum and 1% penicillin/streptomycin, both at 37 °C with 5% CO_2_ (Gibco, Carlsbad, CA, USA).

### 4.12. RNA Extraction and qRT-PCR Analysis 

Total RNA from NCM460 and Caco-2 cells was extracted using the RNAeasy™ Animal RNA Isolation Kit with Spin Column (Beyotime, Shanghai, China), following the manufacturer’s instructions. cDNA for qPCR was synthesized using the HiScript III 1st Strand cDNA Synthesis Kit with gDNA wiper (Vazyme, Nanjing, China), followed by a quantitative real-time PCR assay with Taq Pro Universal SYBR qPCR Master Mix (Vazyme, Nanjing, China). Gene expression was quantified using the 2^−ΔΔCt^ method on a CFX96 Optics Module (785BR20679, BIO-RAD, Hercules, CA, USA), with GAPDH serving as the normalization control.

### 4.13. Immunohistochemistry (IHC) Validation Relying on the HPA Database 

This study’s findings were validated by histochemically scoring IHC maps of human colon tissue samples from the open-access Human Protein Atlas (HPA) database (https://www.proteinatlas.org; accessed on 20 October 2024) through a parallel comparison of normal and cancerous tissue samples. Representative genes from the screened key genes were selected for testing. The formula is as follows: H-score = Σpi(i + 1). In the formula, ‘pi’ represents the percentage of positive cells relative to the total number of cells in the section, while ‘i’ in the equation denotes the coloring intensity. Scoring was determined by the percentage of positive cells, categorized into four grades: 0–25% (1 point), 26–50% (2 points), 51–75% (3 points), and 76–100% (4 points). Cells were evaluated on a 4-point scale for staining intensity: 0 for negative, 1 for weakly positive, 2 for positive, and 3 for strongly positive.

### 4.14. Primers for RT-qPCR 

Differential expression of transcript levels of the three genes CDKN2A, CKMT2,MSLN in the NCM460 cell line and the Caco-2 cell line were examined using RT-qPCR as de-scribed above, using the primers shown in Table 1.

### 4.15. Statistical Analysis

Research in R version 4.3.2 was used for all statistical analyses. To determine statistical dominance, a *p*-value less than 0.05 was used.

## 5. Conclusions

Our comprehensive study of CRGs has identified potential immunotherapy drugs, revealing numerous regulatory mechanisms that influence the TME, immune landscape, clinicopathological features, and prognosis. This will lead to fresh insights into the molecular processes of COAD and improve our ability to treat the condition.

## Figures and Tables

**Table 1:** List of Primers for RT-qPCR.

Transcript	Forward	Reverse
CDKN2A (Human)	GTGTATAGGGTCGGCCATCAA	CCTGCCGTTGTTACCTGAGAG
MSLN (Human)	CCCATTGGACCTGCTGCTATT	CATTGGCCTTCGTGATGCG
CKMT2 (Human)	CCAAGCGCAGACTACCCAG	GGTGTCACCTTGTTGCGAAG
GAPDH (Human)	CAGGAGGCATTGCTGATGAT	GAAGGCTGGGGCTCATTT

## Data Availability

The data from the study can be found for free in online databases or in the paper and its Supplementary Materials.

## Supplementary Materials

The following supporting information can be downloaded at: https://www.mdpi.com/article/10.3390/ijms252111830/s1.
