# Supplementary material for: Comprehensive Analysis and Verification of the Prognostic Significance of Cuproptosis-Related Genes in Colon Adenocarcinoma
Source: Int J Mol Sci. 2024 Nov 4;25(21):11830. doi: 10.3390/ijms252111830 (PMC11546850; doi:10.3390/ijms252111830)
Supplement: Supplementary file 1 [file ijms-25-11830-s001.zip › ijms-3245483-supplementary.pdf]

Supplementary Material

1 Supplementary Figures and Tables

1.1 Supplementary Figures

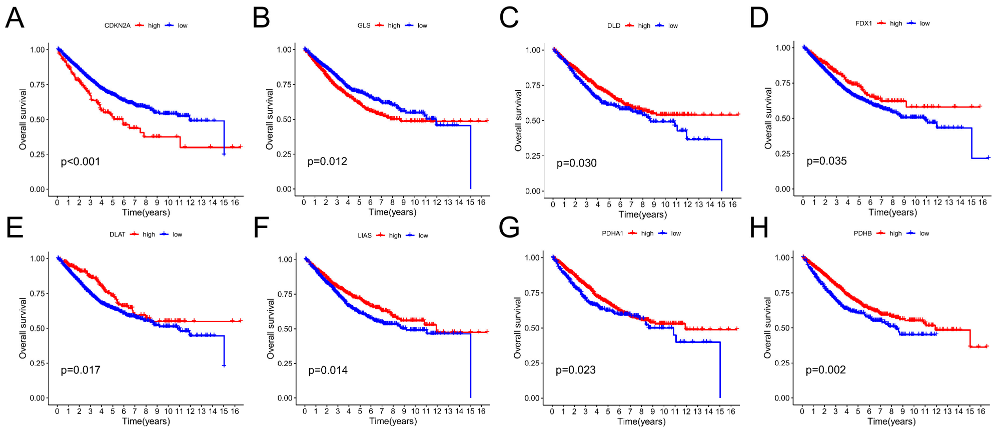

Figure S1. The Kaplan-Meier curves show the survival analysis results of the 8 CRGs. (A) CDKN2A; (B) GLS; (C) DLD; (D) FDX1; (E) DLAT; (F) LIAS; (G) PDHA1; (H) PDHB.

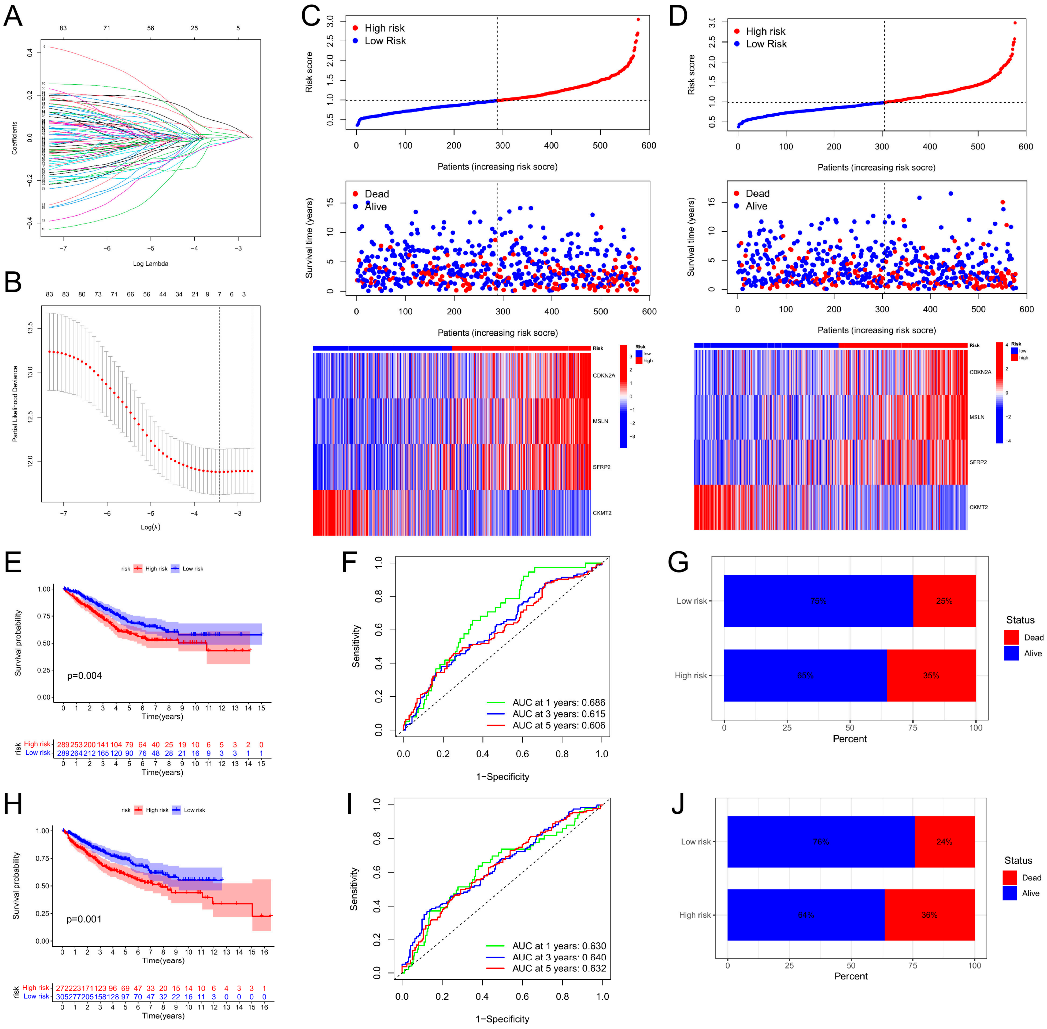

Figure S2. Validation of the prognostic cuproptosis risk score model. (A-B) The Lasso Cox algorithm was used to construct the coefficients for genes with prognostic features; (C-D) Risk distribution, survival status, and related gene expression of cuproptosis risk score in the training set and test sets; (E,H) Comparison of the overall survival rate between the high risk and low risk groupsin the training set and test sets; (F,L) ROC curve of 1-, 3-, 5-year OS of cuproptosis risk score in the training set and test sets; (G,J) OS ratio of patients in the high and low risk groups in the training set and test sets.

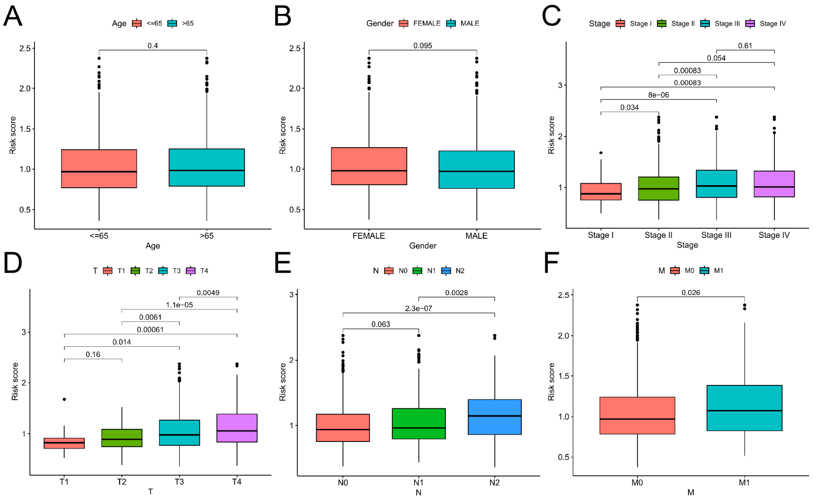

Figure S3. Correlation between cuproptosis risk score and clinical typing. (A) age; (B) gender; (C) stage; (D) T; (E) N; (F) M.

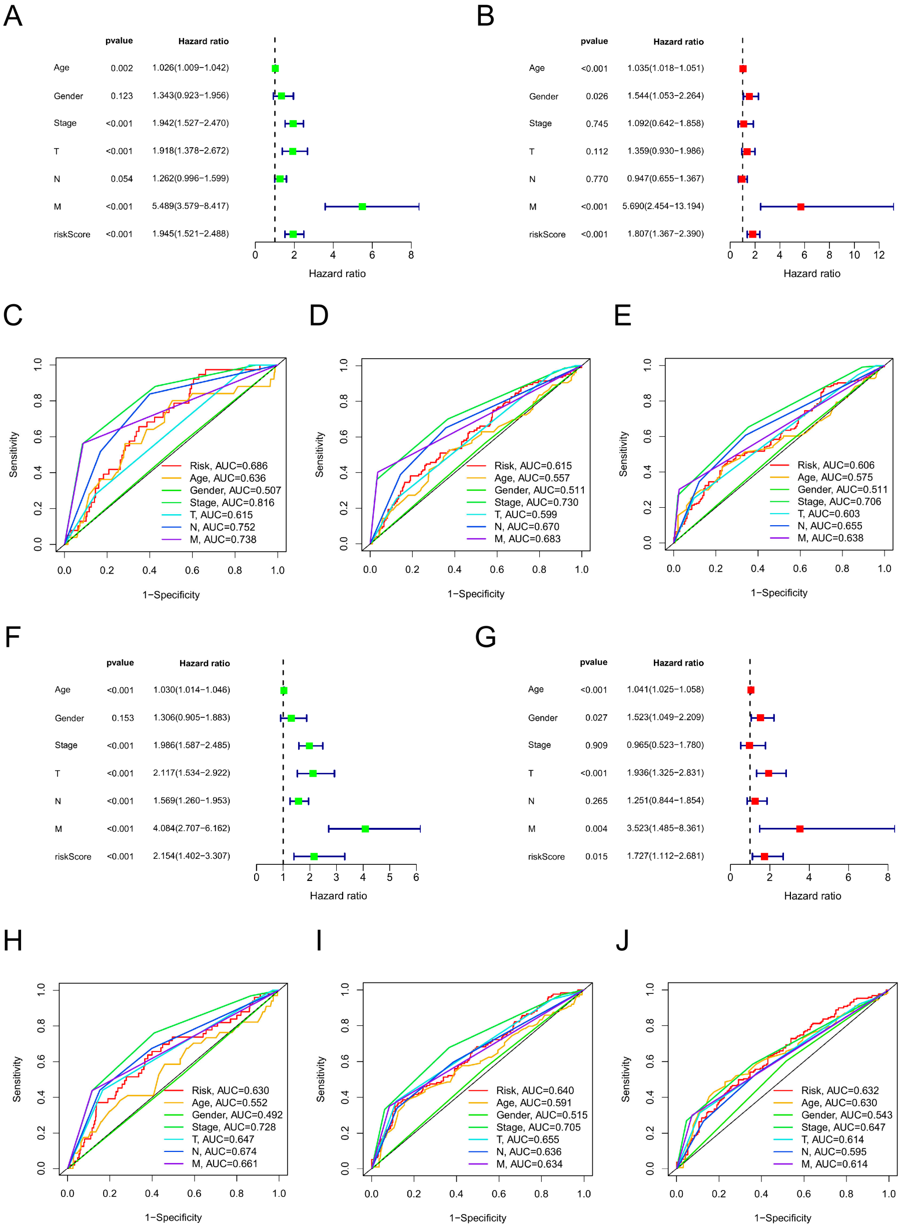

Figure S4. Independent prognostic analysis validation for the training and test sets. (A-B) The training set included univariate and multivariate Cox regression analysis based on cuproptosis risk score and clinicopathological characteristics; (C-E) The ROC curve in the training set evaluated the prediction effect of risk models at 1-, 3-, and 5-year; (F-G) The test set included univariate and multivariate Cox regression analysis based on cuproptosis risk score and clinicopathological characteristics; (H-J) The ROC curve in the test set evaluated the prediction effect of risk models at 1-, 3-, and 5-year.

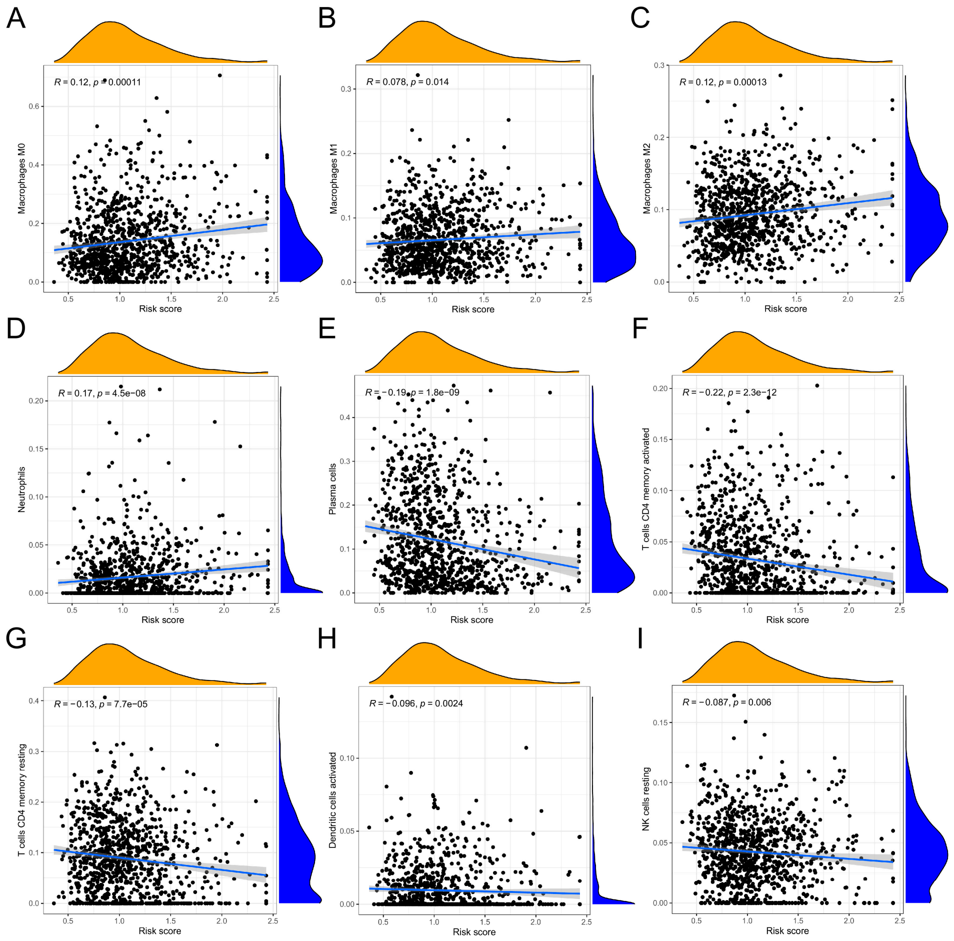

Figure S5. Correlation between cuproptosis risk score and immune cell subtypes. (A) M0 macrophages; (B) M1 macrophages; (C) M2 macrophages; (D) Neutrophils; (E) Plasma cells; (F) CD4 memory activated T cells; (G) CD4 memory resting T cells; (H) Activated dendritic cells; (I) Resting NK cells.

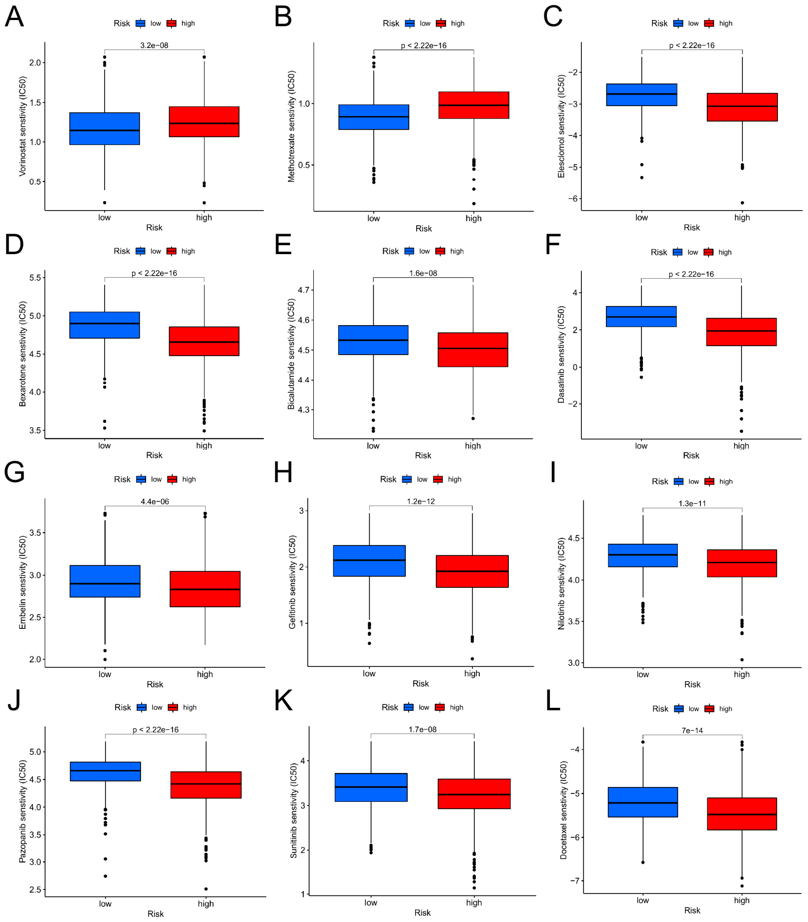

Figure S6. Drug sensitivity analysis of the high and low risk groups. (A) Vorinostat ; (B) Methotrexate; (C) Elesclomol; (D) Bexarotene; (E) Bicalutamide; (F) Dasatinib; (G) Embelin; (H) Gefitinib; (I) Nilotinib; (J) Pazopanib; (K) Sunitinib; (L) Docetaxel.

1.2 Supplementary Tables

Table S1. Risk score coefficients for four genes in constructing the prognostic model.

Gene	Coef	Pvalue
CDKN2A	0.154203547	0.048597778
MSLN	0.086609141	0.022879941

SFRP2	0.058477128	0.000425298
CKMT2	-0.098254951	0.004256958

Table S2. Immune infiltration and function between high and low risk groups in different algorithms.

Immune	Pvalue
T cell CD4+_TIMER	0.01744077
T cell CD8+_TIMER	0.001961653
Neutrophil_TIMER	3.75E-08
Macrophage_TIMER	4.83E-08
Myeloid dendritic cell_TIMER	4.34E-08
B cell plasma_CIBERSORT	1.12E-05
T cell CD4+ memory resting_CIBERSORT	8.22E-06
T cell follicular helper_CIBERSORT	0.000266129
NK cell activated_CIBERSORT	0.028374337
Monocyte_CIBERSORT	0.003468696
Macrophage M0_CIBERSORT	2.83E-06
Macrophage M2_CIBERSORT	0.004323712
Myeloid dendritic cell activated_CIBERSORT	0.000903641
Neutrophil_CIBERSORT	6.10E-05
B cell plasma_CIBERSORT-ABS	0.00695571
T cell CD8+_CIBERSORT-ABS	0.033652003

NK cell activated_CIBERSORT-ABS	9.17E-05
Macrophage M0_CIBERSORT-ABS	1.23E-11
Macrophage M1_CIBERSORT-ABS	0.00039766
Macrophage M2_CIBERSORT-ABS	6.51E-06
Myeloid dendritic cell activated_CIBERSORT-ABS	0.001134731
Neutrophil_CIBERSORT-ABS	1.74E-05
Macrophage M1_QUANTISEQ	0.00818999
Macrophage M2_QUANTISEQ	5.40E-05
Neutrophil_QUANTISEQ	0.031447711
NK cell_QUANTISEQ	0.002689874
T cell CD4+ (non-regulatory)_QUANTISEQ	0.000460101
T cell CD8+_QUANTISEQ	0.00074319
T cell regulatory (Tregs)_QUANTISEQ	0.045773713
Myeloid dendritic cell_QUANTISEQ	0.023305223
T cell CD8+_MCPCOUNTER	0.03591991
cytotoxicity score_MCPCOUNTER	0.00022261
Monocyte_MCPCOUNTER	1.45E-09
Macrophage/Monocyte_MCPCOUNTER	1.45E-09
Myeloid dendritic cell_MCPCOUNTER	0.000160313
Endothelial cell_MCPCOUNTER	2.74E-06

Cancer associated fibroblast_MCPCOUNTER	1.41E-18
Myeloid dendritic cell activated_XCELL	4.24E-05
T cell CD4+ memory_XCELL	0.03946908
T cell CD4+ naive_XCELL	0.01200001
T cell CD8+ effector memory_XCELL	0.016449465
Common lymphoid progenitor_XCELL	0.027850983
Myeloid dendritic cell_XCELL	1.40E-06
Endothelial cell_XCELL	1.53E-06
Hematopoietic stem cell_XCELL	0.001568457
Macrophage_XCELL	1.51E-09
Macrophage M1_XCELL	2.12E-09
Macrophage M2_XCELL	3.87E-07
Mast cell_XCELL	0.043776708
Monocyte_XCELL	2.47E-08
T cell NK_XCELL	0.000154833
Plasmacytoid dendritic cell_XCELL	0.003329478
B cell plasma_XCELL	0.001235365
immune score_XCELL	0.00016082
stroma score_XCELL	1.04E-06
microenvironment score_XCELL	4.53E-06
Cancer associated fibroblast_EPIC	9.21E-18

T cell CD8+_EPIC	0.007762241
Endothelial cell_EPIC	3.54E-07
Macrophage_EPIC	4.32E-11
NK cell_EPIC	1.19E-08
uncharacterized cell_EPIC	6.38E-15
